# Lutein-based dye vitrectomy for idiopathic epiretinal membrane: a pilot study

**DOI:** 10.3389/fmed.2025.1605391

**Published:** 2025-06-16

**Authors:** Settimio Rossi, Carlo Gesualdo, Michele Della Corte, Antonio Del Giudice, Andrea Rosolia, Maria Consiglia Trotta, Francesca Simonelli

**Affiliations:** ^1^Eye Clinic, Multidisciplinary Department of Medical, Surgical and Dental Sciences, University of Campania “Luigi Vanvitelli”, Naples, Italy; ^2^Department of Experimental Medicine, University of Campania "Luigi Vanvitelli", Naples, Italy

**Keywords:** chromovitrectomy, vitreoretinal surgery, vitreous body staining, posterior hyaloid detachment, idiopathic epiretinal membrane

## Abstract

**Introduction:**

The use of vital dyes is essential for enhancing visualization of the vitreous and vitreoretinal interface during surgery. In this study, we evaluated the efficacy of a new lutein-based vital dye (LB-VD) for selective staining of the vitreous body and posterior hyaloid in patients undergoing 25 G vitrectomy for idiopathic epiretinal membrane (ERM).

**Methods:**

A total of 18 patients who underwent ERM surgery assisted by LB-VD were retrospectively analyzed. The surgeon completed a postoperative satisfaction questionnaire to evaluate the vitreous staining. Data on best-corrected visual acuity (BCVA), central retinal thickness (CRT), intraocular pressure (IOP), and fixation stability were collected at baseline and at 4 and 6 months post-surgery.

**Results:**

LB-VD effectively stained the vitreous body and posterior hyaloid, facilitating key surgical maneuvers. Significant improvements were observed in BCVA and CRT at both 4 and 6 months post-surgery. Specifically, BCVA improved from 0.60 ± 0.29 LogMAR at baseline to 0.33 ± 0.26 LogMAR at 4 months (*p* = 0.004) and to 0.32 ± 0.25 LogMAR at 6 months (*p* = 0.003). CRT decreased significantly from 429 (375–499) μm at baseline to 335 (254–375) μm at 4 months (*p* = 0.03) and to 323 (240–356) μm at 6 months (*p* = 0.01). Fixation stability improved in approximately 95% of patients by 6 months. No significant changes in IOP were noted.

**Discussion:**

LB-VD provided clear visualization of the vitreous body and posterior hyaloid, thereby facilitating key vitreoretinal surgical tasks. These findings suggest that LB-VD is an effective and safe alternative to traditional dyes currently used in vitreoretinal surgery.

## 1 Introduction

The ability to visualize tissues and anatomical planes is crucial in vitreoretinal surgery. To improve visualization of the vitreous, internal limiting membrane (ILM), and the epiretinal membrane and to enable delicate maneuvers, vital dyes or crystals are used to stain these intraocular components (1, 2).

Vital dyes are molecules containing chromophores that bind living tissues through chemical reactions, thereby enhancing visualization and their subsequent removal during vitreoretinal surgery (2). The first vital dye introduced for this purpose was indocyanine green (ICG), which was used to improve the visualization of the ILM. However, numerous clinical and experimental studies have revealed retinal toxicity associated with ICG, including changes in the retinal pigment epithelium (RPE), visual field defects, and optic nerve atrophy. These challenges have led to the exploration of alternative dyes, such as Trypan Blue (TB), Patent Blue (PB), and Brilliant Blue G (BBG) (3–6).

In this regard, triamcinolone acetonide (TA) is traditionally used to stain the vitreous, aiding in visualization and delicate surgical maneuvers such as performing posterior vitreous detachment, cutting the vitreous base, and removing the peripheral vitreous cortex. Unlike vital dyes, TA adheres to acellular tissues and consists of water-insoluble synthetic corticosteroid crystals that connect vitreous fibrils, enhancing visualization (7). Two formulations of TA are used in vitreoretinal surgery: non-preservative-free triamcinolone acetonide (non-PFTA) (Kenalog^®^; Bristol-Myers Squibb, NJ), which contains 0.99% benzyl alcohol as a preservative, and preservative-free triamcinolone acetonide (PFTA; Triesence^®^; Alcon, Fort Worth, Texas). Initially used as an off-label intravitreal treatment of macular edema, TA has been adopted as a dye in pars plana vitrectomy (PPV) (8). However, studies have shown increased septic risk, higher post-injection intraocular pressure, and toxicity to human neuroretinal and RPE cell cultures, primarily due to the presence of benzyl alcohol (9–14). Therefore, several strategies have been adopted to reduce the potential toxicity of TA, such as dilution with a balanced salt solution. Finally, PFTA formulations were developed and approved for intravitreal use; however, even in these formulations, the results regarding the safety profile remain controversial (15–18). Furthermore, TA is a non-selective dye that stains indiscriminately all tissues at the vitreous–retinal interface, including the vitreous, posterior hyaloid, vitreous cortex residues, ERM, and ILM (2, 7).

The introduction of a lutein-based vitreous dye (LB-VD; Vitreo Lutein) offers a potentially safer alternative to TA-based formulations. LB-VD not only improves visualization of vitreous fibrils but also provides additional benefits related to blue light filtering and the neuroprotective and antioxidant properties of lutein and zeaxanthin (19, 20). *In vitro*, LB-VD demonstrated a superior safety profile over all TA-based formulations in terms of cell vitality, metabolism, and proliferation (15). Moreover, LB-VD selectively stains the vitreous and posterior hyaloid without interacting with the membranes at the vitreous–retinal interface (1, 2).

To date, no clinical studies have evaluated the safety and efficacy of LB-VD. Therefore, this study aims to assess the properties of LB-VD in a cohort of patients with idiopathic ERM undergoing PPV.

## 2 Materials and methods

A retrospective observational study was conducted at the Eye Clinic of the University of Campania “Luigi Vanvitelli,” involving 18 patients who underwent vitrectomy for idiopathic macular pucker. The study adhered to the principles outlined in the Declaration of Helsinki and was approved by the Ethics Committee of the University of Campania “Luigi Vanvitelli” (Protocol 0013692/I, 09 May 2024). All participants were informed about the potential risks and benefits of the surgical procedure, and they consented to participate by signing a written informed consent form.

Patients with idiopathic ERM were included in the study. The exclusion criteria were as follows: refractive error ≥ ± 6.0 diopters, history of ocular trauma or surgery, advanced cataract, glaucoma or other optic neuropathies, secondary epiretinal membrane (ERM), choroidal neovascularization of any origin, diabetic retinopathy, retinal vascular occlusion, central serous chorioretinopathy (CSCR), macular hole, macular telangiectasia, and age-related macular degeneration (AMD).

Baseline data, including gender, age, best-corrected visual acuity (BCVA), central retinal thickness (CRT), intraocular pressure (IOP), microperimetry (MP), and lens status (phakic and pseudophakic), are summarized in Table 1.

**Table 1:** Baseline demographic and ocular characteristics.

Total number of patients analyzed, *n*	18
Sex, *n* (%)	
Male	7 (38,8)
Female	11 (61,2)
Age, years	
Mean ± SD	69,77 ± 5,15
BVCA (LogMAR)	
Mean ± SD	0.33 ± 0.26
CRT, μm	
Median (IQR)	429 (375–499)
IOP, mmHg	
Median (IQR)	14 (13–16)
Microperimetry sensitivity, dB	
Median (IQR)	14 (12–16)
Fixation stability, *n* (%)	
Stable	7 (39)
Relatively stable	1 (5)
Unstable	10 (56)
Lens status, *n* (%)	
Phakia	6 (30)
IOL	12 (70)

BCVA: best-corrected visual acuity; CRT: central retinal thickness; IOP: intraocular pressure; MS: macular sensitivity; FS: fixation stability.

All surgeries were performed by the same expert vitreoretinal surgeon (SR), using the vital dye LB-VD (crystallin lutein 2%; 019004 Vitreo Lutein™; VitreoCare – Alfa Instruments, Italy) to visualize the vitreous body during 25G PPV. In phakic patients, vitrectomy was combined with phacoemulsification of the cataract and implantation of an intraocular lens (IOL) to prevent cataract formation from affecting the final visual outcome. The surgical procedure included the following steps: performing three sclerotomies via 25G trocars; initial vitrectomy; introduction of LB-VD; core vitrectomy with removal of the posterior hyaloid; introduction of Dual blue dye with a 2 min wait time; epiretinal membrane peeling; inspection of the peripheral retina; air tamponade; and suturing of sclerotomies, if necessary. An intraoperative OCT visualization system (iOCT-ZEISS ARTEVO 800) was employed throughout the procedure to facilitate visualization of the vitreous body and to assess the outcome of epiretinal membrane peeling. All procedures adhered to international standards, and no intraoperative or postoperative complications were reported. Surgical procedures were recorded in MP4 format, enabling frame extraction for the analysis of LB-VD’s effectiveness in optimizing vitreous and posterior hyaloid visualization. Additionally, at the end of each vitrectomy, the surgeon completed a satisfaction questionnaire to evaluate LB-VD’s ability to distinctly identify the vitreous body and posterior hyaloid.

All patients underwent a comprehensive ophthalmological examination, which included measuring the best-corrected visual acuity (BCVA-LogMAR) using Early Treatment Diabetic Retinopathy Study (ETDRS) charts at 2 m, assessing the anterior segment with slit lamp biomicroscopy, and measuring intraocular pressure with Goldmann applanation tonometry. Fundus examination was conducted using binocular indirect ophthalmoscopy, and spectral domain optical coherence tomography (SD-OCT) was performed using the ZEISS CIRRUS 5000 system (Carl Zeiss, Dublin, CA), using a five-line raster scan and macular cube scan pattern. Additionally, microperimetry was performed with the Nidek MP-3 (Nidek Technologies, Italy) to evaluate the mean sensitivity of the central macular area and fixation stability within central 2 and 4 degrees (FS 2° and FS 4°).

These examinations were repeated at baseline, 4 months, and 6 months after vitrectomy to assess anatomical and functional changes resulting from the surgery.

### 2.1 Statistical analysis

The Shapiro–Wilk test and Levene’s test were employed to assess data distribution and homogeneity. Variables based on repeated observations were analyzed using Friedman’s test, followed by Dunn’s *post-hoc* test, or using repeated measures analysis of variance (RM-ANOVA), followed by Bonferroni’s multiple comparison test. For RM-ANOVA, Mauchly’s test was performed to assess sphericity, and if violated, the appropriate correction (Greenhouse–Geisser or Huynh-Feldt) was applied. RM-ANOVA data were reported as mean ± standard deviation (SD), while skewed data were presented as median (interquartile range -IQR). A *p*-value of <0.05 was considered statistically significant.

## 3 Results

At the Eye Clinic of the “Luigi Vanvitelli” University of Campania, 18 patients (7 men and 11 women) with a mean age of 69.8 ± 5 years, diagnosed with idiopatic macular pucker and undergoing 25G vitrectomy, were enrolled. A total of 18 eyes (one from each patient) were analyzed. The baseline informations are summarized in Table 1.

**Figure 1:**
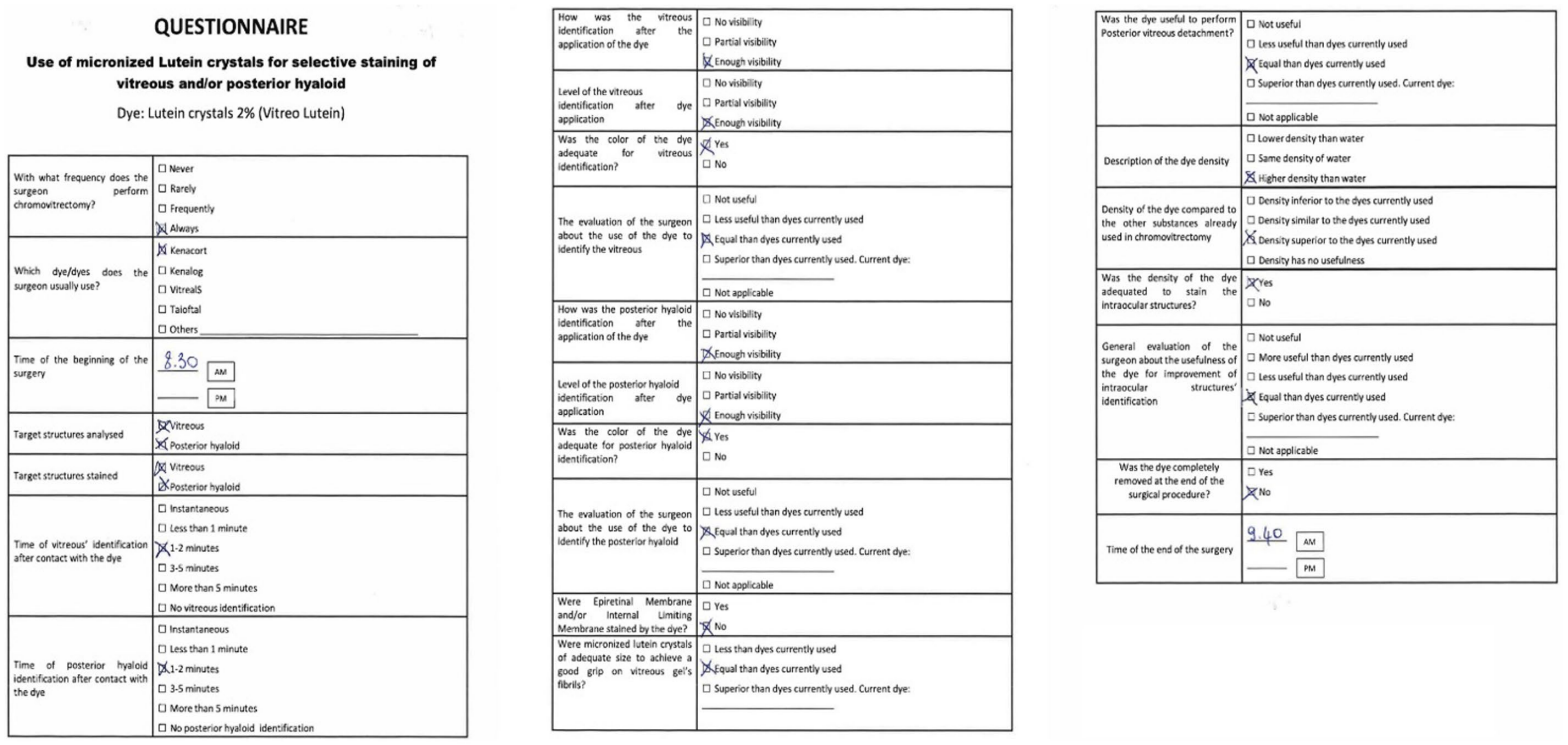
Questionnaire on the use of micronized lutein crystals for selective staining of the vitreous and/or posterior hyaloid during PPV. The form includes questions regarding the frequency of dye use, time of surgery, identification of target structures, and the surgeon’s evaluation of the dye’s usefulness for surgical procedures such as posterior vitreous detachment.

**Figure 2:**
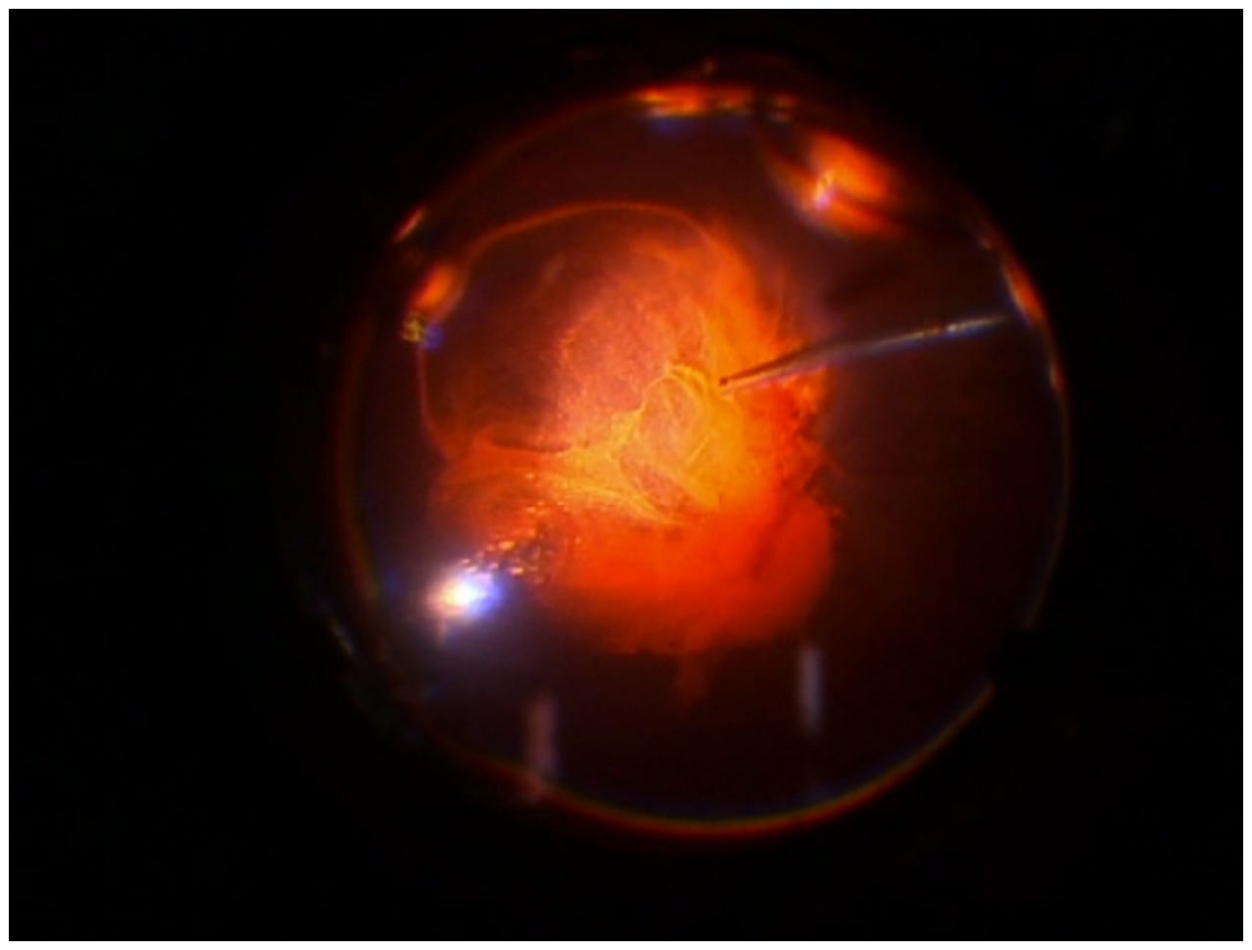
Intraoperative image demonstrating successful staining and ability to highlight the vitreous and the posterior hyaloid with LB-VD.

All surgical procedures were performed according to international standards, and no intra-operative and/or postoperative complications, inflammation, or adverse events were recorded. After the procedure, the surgeon completed a satisfaction questionnaire and expressed satisfaction with the LB-VD dye’s ability to distinctly identify the vitreous body and posterior hyaloid (Figure 1). Additionally, by extracting frames from the surgical videos, the dye’s effective performance was objectively documented. LB-VD was observed to integrate well with the vitreous gel, selectively coloring the posterior hyaloid due to its specific affinity (Figure 2).

**Figure 3:**
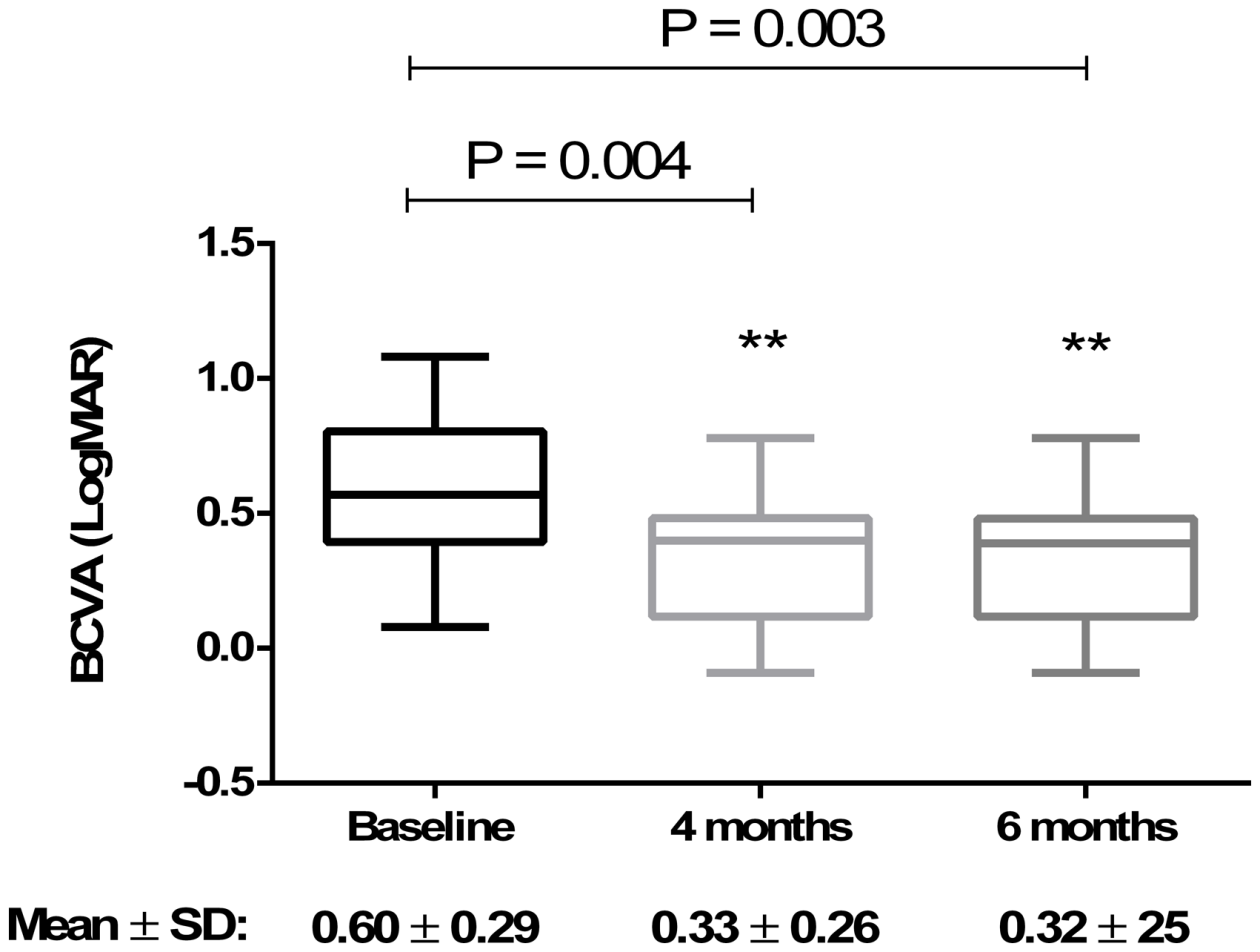
Improvement in best-corrected visual acuity (BCVA) at 4 and 6 months compared to baseline. The box plot shows a significant increase (*p* < 0.01) in mean BCVA at 4 months (0.33 ± 0.26 LogMAR) and 6 months (0.32 ± 0.25 LogMAR) compared to baseline (0.60 ± 0.29 LogMAR) (RM-ANOVA followed by Bonferroni’s test and Geisser–Greenhouse correction). Data are reported as mean ± standard deviation (SD).

**Figure 4:**
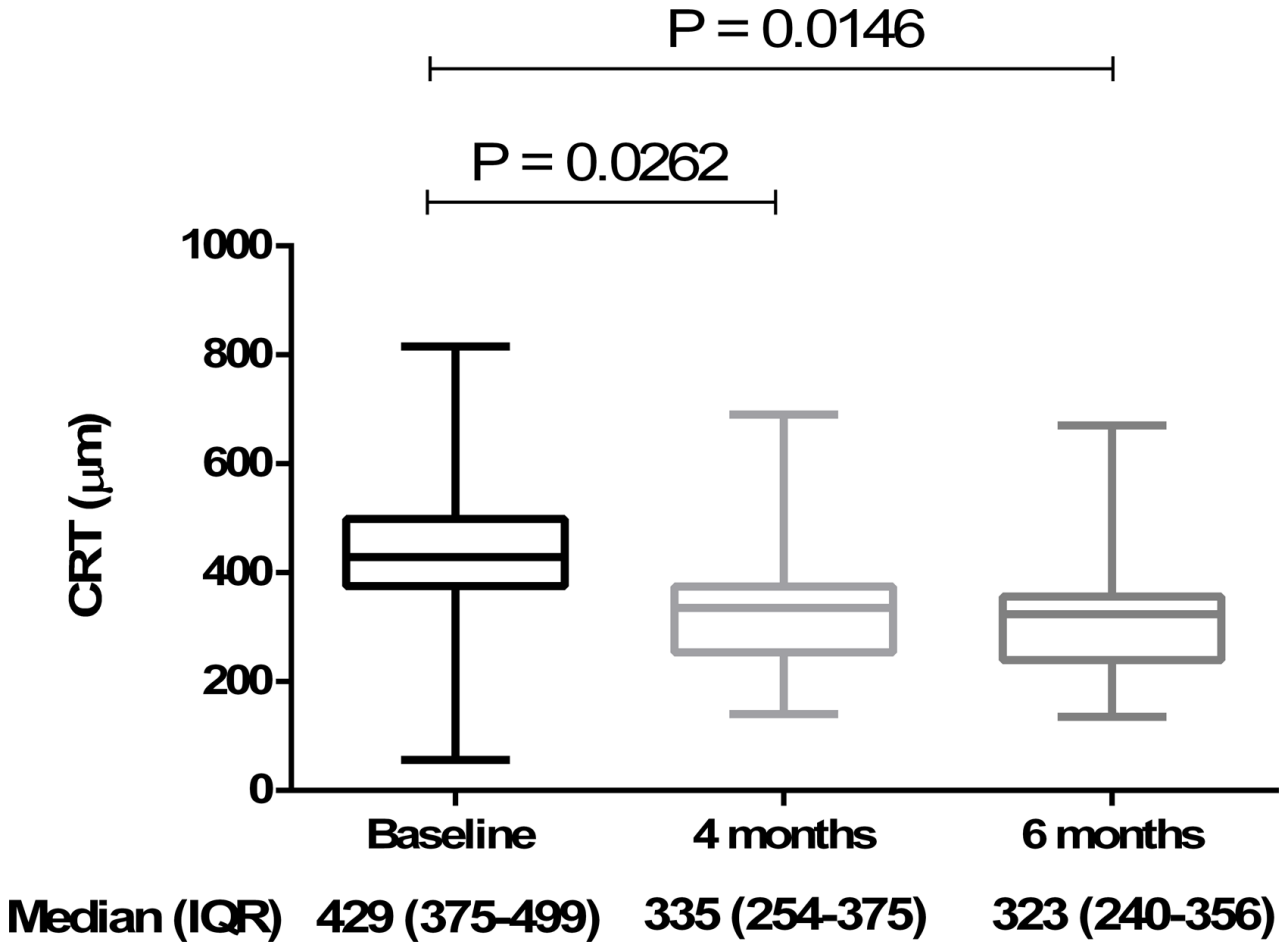
Changes in central retinal thickness (CRT) at 4 and 6 months compared to baseline. The box plot shows a significant reduction (*p* < 0.05) in CRT at 4 months [335 (254–375) μm] and 6 months [323 (240–356) μm] compared to baseline [429 (375–499) μm] (Friedman’s test, followed by Dunn’s test). Data are reported as median and interquartile range (IQR).

**Figure 5:**
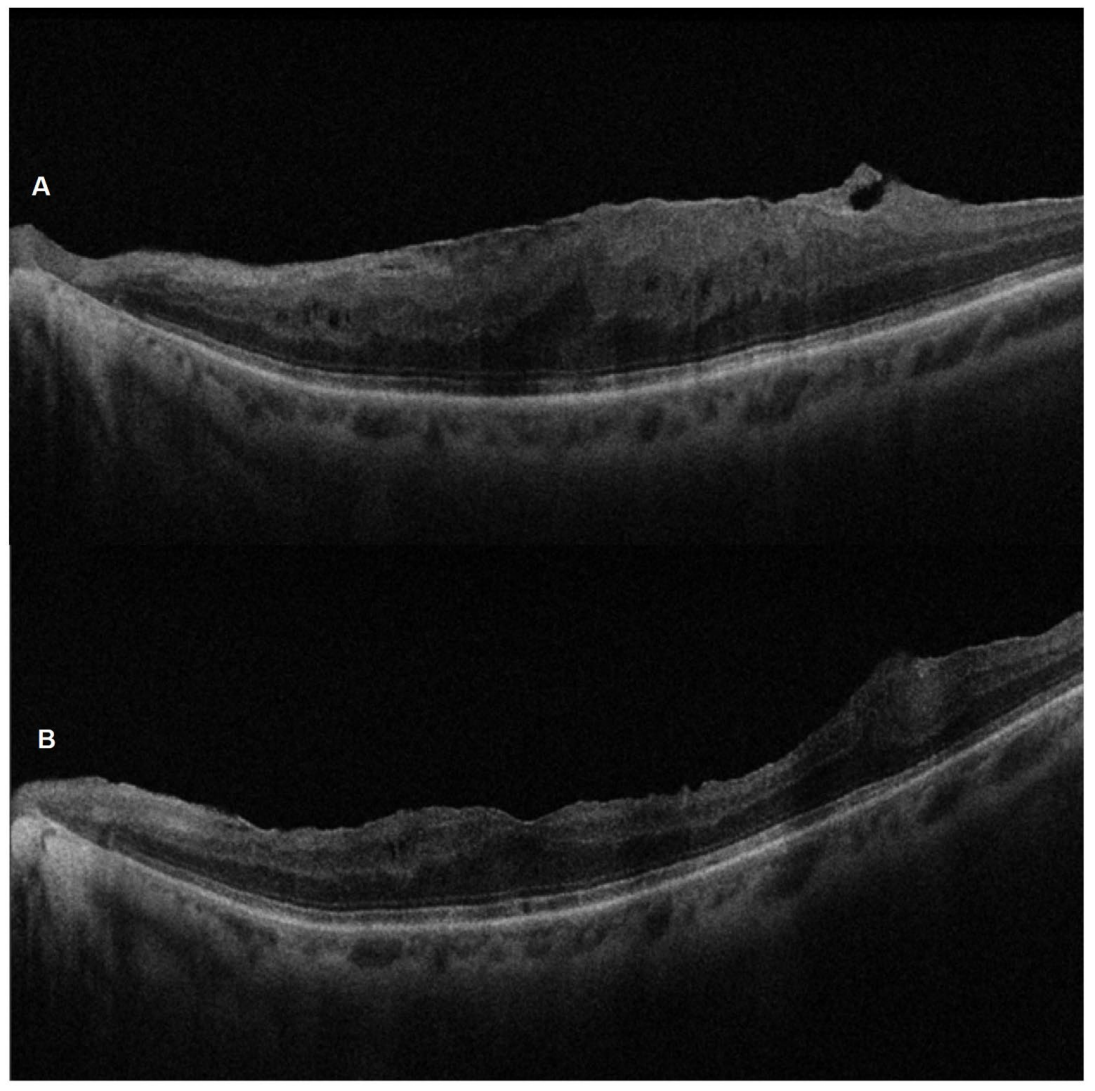
SD-OCT pre-operative **(A)** and 6-month post-ERM surgery **(B)** images of the left eye of a 67-year-old woman. Note the successful peeling and the morphological recovery of the foveal profile.

**Figure 6:**
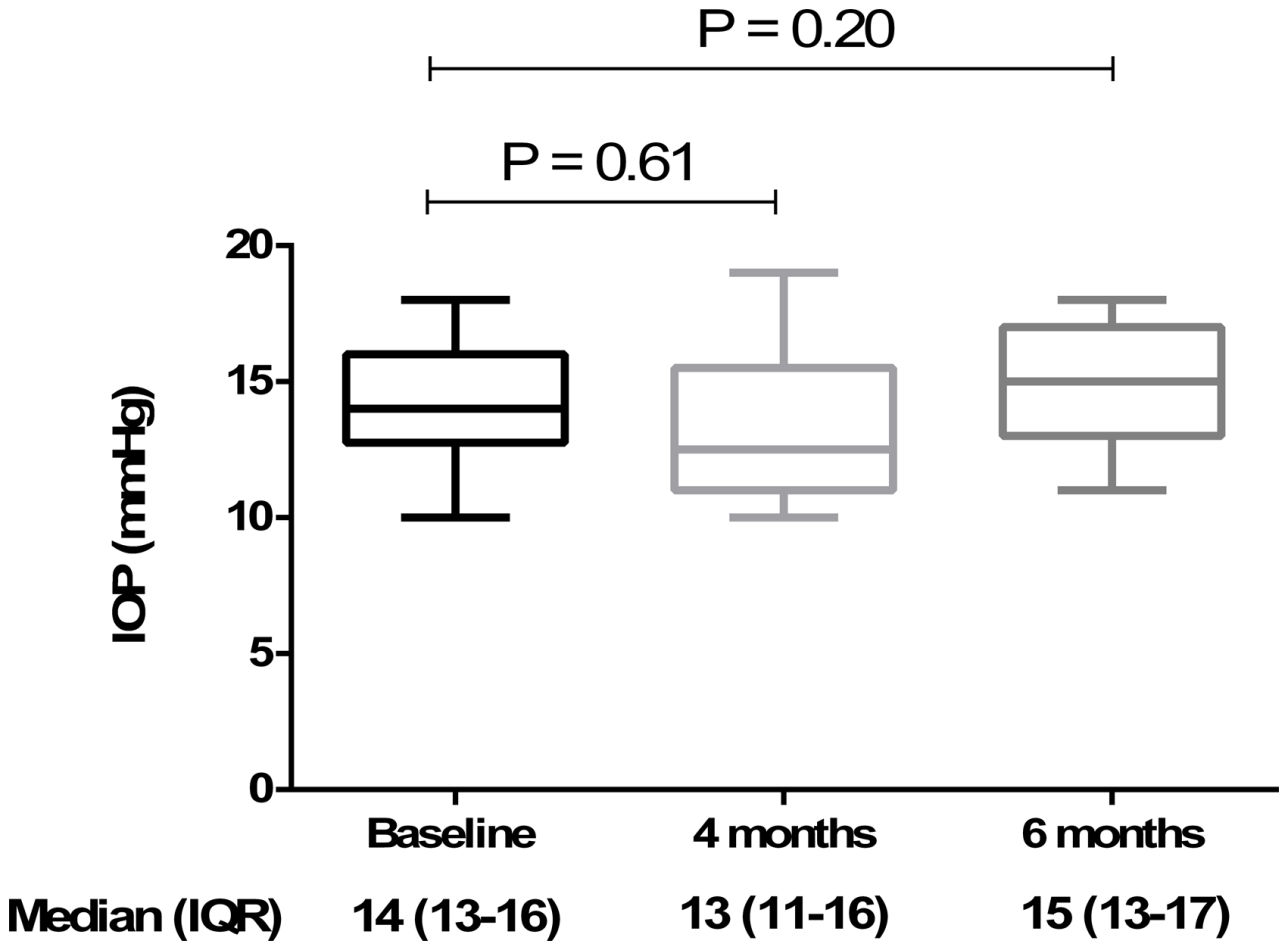
Analysis of intraocular pressure (IOP) at baseline, 4 months, and 6 months. The box plot shows stable IOP values across all time points, with no significant differences (*p* > 0.05) at 4 months [13 (11–16) mmHg] and 6 months [15 (13–17) mmHg] compared to baseline [14 (13–16) mmHg] (Friedman’s test, followed by Dunn’s test). Data are reported as median and interquartile range (IQR).

**Figure 7:**
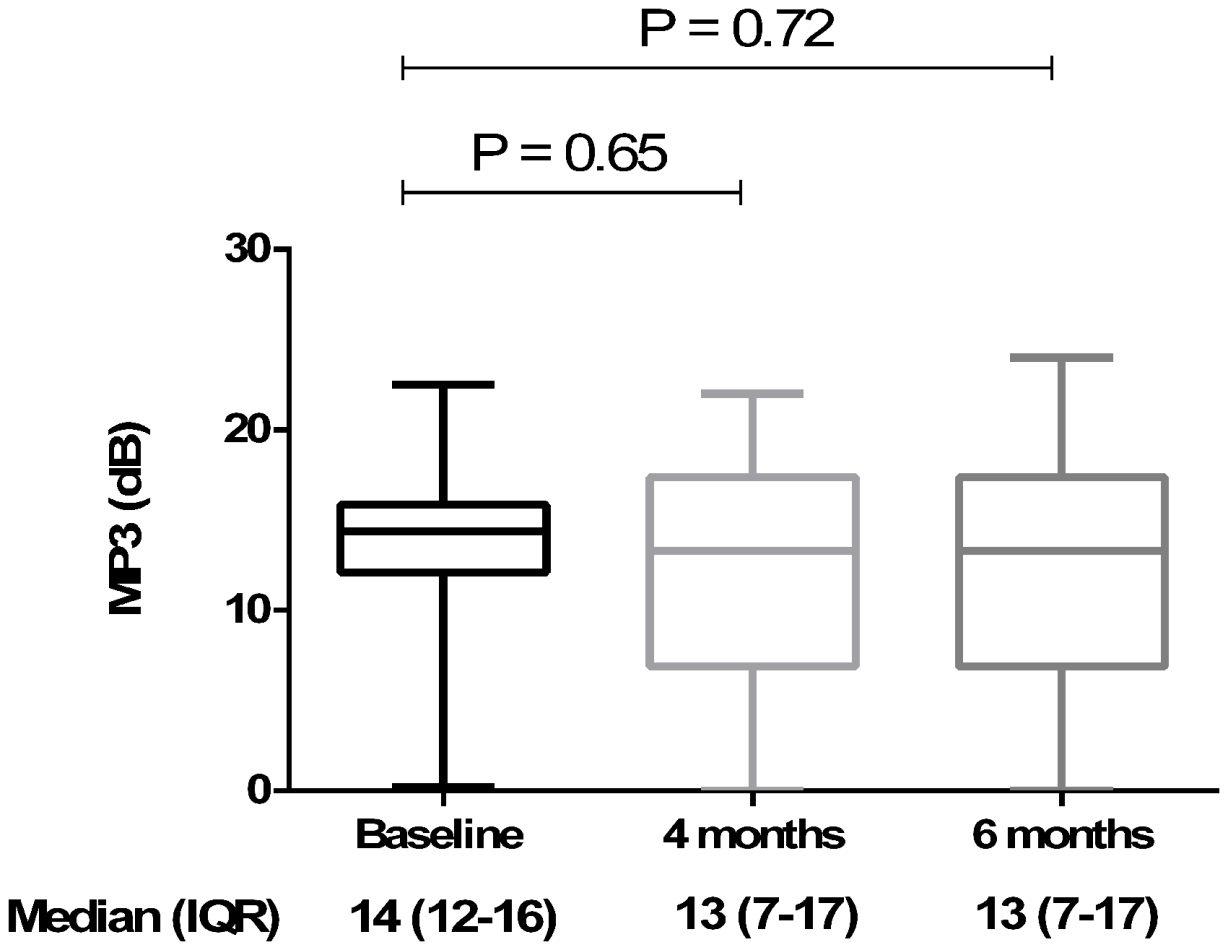
Retinal sensitivity measured using microperimetry (MP3) at 4 and 6 months compared to baseline. The box plot shows stable MP3 over the time: 14 (12–16) dB at baseline, 13 (7–17) dB at 4 months, and 13 ± (7–17) dB at 6 months. No statistically significant differences (*p* > 0.05) were observed at 4 and 6 months (compared to baseline) (Friedman’s test, followed by Dunn’s test). Data are reported as median and interquartile range (IQR).

Analyzing the functional results after vitrectomy, we observed that all patients exhibited a statistically significant improvement in BCVA at 4- and 6-month follow-ups. At 4 months, the average BCVA improved from 0.60 ± 0.29 LogMAR to 0.33 ± 0.26 LogMAR (*p* = 0.004). At 6 months, it further improved to 0.32 ± 0.25 LogMAR (*p* = 0.003). No significant difference in BCVA was observed between the 4- and 6-month time points (*p* > 0.05) (Figure 3). A significant reduction in mean central retinal thickness (CRT) was observed both at 4- and 6-month post-surgery. At 4 months, the mean CRT decreased from 429 (375–499) μm to 335 (254–375) μm (*p* = 0.03). At 6 months, the CRT was further reduced to 323 (240–356) μm (*p* = 0.01). No significant differences in CRT were noted between the 4-month and 6-month follow-up periods (*p* > 0.05) (Figures 4, 5). Analysis of intraocular pressure (IOP) revealed no significant changes compared to baseline during the follow-up period (*p* > 0.05) (Figure 6). Microperimetry results revealed no significant changes in macular sensitivity compared to baseline at any follow-up time point (*p* > 0.05). However, with regard to fixation stability, the following observations were recorded: (a) 56% of patients (*n* = 10) who had unstable fixation at baseline achieved stable fixation by 6 months after vitrectomy; (b) 39% of patients (*n* = 7) maintained stable fixation throughout the follow-up period; and (c) 5% of patients (*n* = 1) had unstable fixation both at baseline and at all follow-up time points (Figure 7).

## 4 Discussion

The evaluation of the efficacy and safety of various dyes used in vitreoretinal surgery remains a critical and evolving area of research. In this context, we have conducted an innovative analysis of a new LB-VD designed specifically for selective staining of the vitreous body and posterior hyaloid. This study focused on a cohort of patients with idiopathic ERM who underwent 25G PPV with LB-VD assistance.

Since 2000, chromovitrectomy, the intraocular application of dyes to facilitate visualization of preretinal tissues during vitreoretinal surgery, has become a popular approach to facilitate the performance of key surgical maneuvers, such as ILM and ERM peeling, along with sufficient vitreous removal. The first dye used in chromovitrectomy, ICG, facilitated ILM identification but showed multiple signs of retinal toxicity (5, 6). Subsequently, additional dyes, such as TB and PB, were introduced for the identification of the ERM, and TA for a better vitreous visualization. Finally, more recently, other natural dyes with anti-inflammatory and antioxidant properties, such as lutein and anthocyanin derived from the açaí fruit, have been proposed (21–24).

TA has traditionally served as the reference dye in vitreoretinal surgery due to its effectiveness in staining the vitreous gel, thereby enhancing visualization and facilitating delicate surgical maneuvers such as posterior vitreous detachment and removal of vitreous cortex remnants (7). Despite the introduction of various formulations of TA, including PFTA versions and injectable suspensions (e.g., Triesence), concerns about its safety persist. Challenges such as cytotoxicity, IOP elevation, cataract development, and risk of sterile endophthalmitis are notable (16, 25–30). Preclinical studies have highlighted that non-PFTA formulations, primarily due to the presence of benzyl alcohol, can lead to reduced cell survival, apoptosis, and subsequent retinal degeneration in human retinal cell cultures (14, 31–34). To address these concerns, several strategies have been employed, including dilution with a balanced salt solution and the development of preservative-free formulations. In recent times, the preservative-free triamcinolone acetonide injectable suspension (TAIS; TRIESENCE; Alcon, Inc., Fort Worth, TX) has been introduced (35); however, even with this latest formulation, questions about retinal cytotoxicity remain. For instance, research by Spitzer et al. has shown that PFTA formulations tend to form larger crystalline aggregates, which can increase contact with retinal tissue and pose a higher risk of cytotoxicity (18).

In recent times, other natural dyes, such as lutein and anthocyanin derived from açaí fruit, have been proposed for intraocular application during vitrectomy. In particular, a new lutein-based vital dye has been introduced as a potentially safer alternative (36). Using *in vitro* models, Lazzara et al. recently demonstrated that LB-VD showed a better safety profile than all TA-based formulations, even when used in combination with perfluorodecalin (PFD), showing the highest levels of cell viability, metabolism, and proliferation (15). Furthermore, LB-VD offers advantages linked to its antioxidant properties, neuroprotective effects, and blue light filtering action. These benefits are attributable to the presence of lipophilic pigments, such as lutein and zeaxanthin, which are physiologically present in the retina.

Furthermore, some authors have highlighted the ability of crystallized lutein-based dyes to improve the visualization of epiretinal membranes using iOCT compared to soluble lutein-based formulations (37). Although lutein-based blue dyes have already been used in vitreoretinal surgery to stain the ILM and/or ERM, demonstrating a good safety profile and effective staining capabilities (36, 38), no clinical studies, to date, have evaluated the properties of crystallin lutein 2% LB-VD. Therefore, we analyzed the effectiveness of LB-VD in staining and improving the visualization of the vitreous body and the posterior hyaloid. We also evaluated the anatomical and functional changes in a cohort of 18 patients with idiopathic macular pucker who underwent 25G PPV assisted by LB-VD. The results were promising: the surgeon reported high levels of satisfaction with LB-VD (as demonstrated by the outcome of the questionnaires), highlighting its excellent ability to visualize the vitreous and the posterior hyaloid, which turned an orange color shortly after dye injection. This facilitated key surgical maneuvers, such as posterior hyaloid detachment and removal of residual peripheral vitreous cortex. Additionally, we observed statistically significant improvements in BCVA and CRT both at 4- and 6-month post-vitrectomy. Fixation stabilization was achieved in approximately 95% of patients by the end of the 6-month follow-up, with no significant changes in intraocular pressure.

## 5 Conclusion

In conclusion, despite certain study limitations, such as a small sample size, short follow-up, and a collection of questionnaire responses obtained from a single surgeon, our results support the hypothesis that LB-VD is comparable to various vitreous dye formulations, such as TA, in determining effective staining of the vitreous and the posterior hyaloid, further allowing for better ERM visualization through the iOCT. Furthermore, LB-VD shows a specific selectivity for the vitreous body and the posterior hyaloid without any risk of intraoperative or postoperative complications. Overall, these findings suggest that LB-VD may offer a valuable and safer alternative to traditional dyes used in vitreoretinal surgery, with the added benefit of potentially reducing cytotoxic risks while improving surgical outcomes. Further studies are warranted to validate these findings and explore the long-term benefits and safety of LB-VD in a broader patient population.

## Data Availability

The original contributions presented in the study are included in the article/supplementary material, further inquiries can be directed to the corresponding author.
